# Bisphenol A in the Urine: Association with Urinary Creatinine, Impaired Kidney Function, Use of Plastic Food and Beverage Storage Products but Not with Serum Anti-Müllerian Hormone in Ovarian Malignancies

**DOI:** 10.3390/ijms26104811

**Published:** 2025-05-17

**Authors:** Mateja Sladič, Špela Smrkolj, Gorazd Kavšek, Senka Imamovic-Kumalic, Ivan Verdenik, Irma Virant-Klun

**Affiliations:** 1 Division of Gynaecology and Obstetrics, University Medical Centre, 1000 Ljubljana, Slovenia; mateja.sladic@kclj.si (M.S.); spela.smrkolj@mf.uni-lj.si (Š.S.); gorazd.kavsek@kclj.si (G.K.); senka.imamovic.kumalic@kclj.si (S.I.-K.); ivan.verdenik@guest.arnes.si (I.V.); 2 Medical Faculty, University of Ljubljana, 1000 Ljubljana, Slovenia; 3 Clinical Research Centre, University Medical Centre Ljubljana, 1000 Ljubljana, Slovenia

**Keywords:** anti-Müllerian hormone, bisphenol A, borderline ovarian tumor, female exposure, kidney function, lifestyle habits, ovarian cancer, thrombocytes, urine

## Abstract

Bisphenol A (BPA) is a high-production-volume industrial chemical and component of commonly used plastic products. However, it is also an endocrine-disrupting chemical that can negatively affect human health. It is not yet known whether it is associated with the development of epithelial ovarian cancer (EOC), a severe and highly fatal human disease. Therefore, the purpose of this study was to determine the concentrations of BPA in the urine of women with EOC or epithelial borderline ovarian tumors (EBOTs) using gas chromatography tandem mass spectrometry (GC-MS/MS) and find their possible associations with kidney function at the molecular level, urine and blood biochemical parameters related to metabolism, anti-Müllerian hormone (AMH) (a marker of ovarian reserve/fertility), and lifestyle habits determined via a questionnaire in comparison to healthy controls. The results suggest that the unadjusted or urine-specific-gravity-adjusted BPA levels were significantly increased in women with EOC/EBOT. The unadjusted BPA was significantly positively associated with urinary creatinine (*p* = 0.007) in all women with EOC/EBOT after adjustment for age, body mass index, and pregnancy using multiple linear regression analysis. This may be related to kidney injury. However, no association was found between urinary BPA and serum AMH levels in women. Women with ovarian malignancies were more exposed to plastic products for storing foods and drinks. Some lifestyle habits, including refilling plastic bottles, correlate with higher urinary BPA levels across the entire cohort of women. When considering EOC or EBOT, it is necessary to consider the potential higher exposure of women to BPA, as reflected in their urine and lifestyle habits.

## 1. Introduction

Bisphenol A (BPA) is a high-production-volume industrial chemical that is widely used in the manufacture of polymer products such as polycarbonates, epoxy resins, polyester resins, polysulfone, and polyacrylate [1,2,3]. Additionally, BPA is used as a color developer in the production of thermal paper, as an antioxidant and inhibitor of end polymerization in polyvinyl chloride (PVC) plastics, and as a precursor for the synthesis of the flame retardant tetrabromobisphenol-A [1,3,4]. Due to its resistance to high temperature and acids, hardness, strength, and transparency, polycarbonates are extensively used in a variety of products such as water bottles, infant feeding bottles, toys, tableware, storage containers, sports equipment, and medical devices [5,6]. Epoxy resins have the capacity to endure heat and chemical substances; therefore, they are used as internal protective linings for food and beverage cans, paints, and adhesives [3,5]. BPA is also present in some resin-based composite filling materials, sealants, and bonding materials used in dentistry [7].

Chemically, BPA is defined by the International Union of Pure and Applied Chemistry (IUPAC) name of 4-[2-(4-hydroxyphenyl) propane-2yl] phenol and is a white, crystalline solid substance that is highly soluble in fats and almost insoluble in water [8,9]. BPA is defined as an endocrine-disrupting chemical (EDC) and was added by the European Chemical Agency (ECHA) Member State Committee (MSC) to the candidate list of substances of very high concern (SVHC) in 2017 [10]. BPA is also classified by the Classification, Labeling, and Packaging (CLP) Regulation ((EC) No. 1272/2008) as a substance that may be hazardous to the reproductive system as well as having the capacity to cause skin allergies and respiratory and eye irritation [8]. During manufacturing, BPA is melted at elevated temperatures, and its particulates are released through effluent to the environment. Consequently, BPA is present in the air, soil, sediment, streams, rivers, drinking water, and wildlife [5,11]. Routes of BPA exposure in humans include both dietary (food containers, take-away water bottles, inner coatings for food cans, microwaveable utensils, etc.) and non-dietary sources (electronics, medical and sport equipment, paints and printing inks, thermal paper, flame retardants, dental materials, DVDs, etc.), with environment contamination directly or indirectly affecting humans [8,12]. Dietary exposure presents the primary route, which includes the ingestion of BPA-polluted seafood and freshwater fish, groceries from polluted areas, and the consumption of food and drinks from plastic containers/bottles [12]. Dermal exposure is the second-leading route of BPA exposure [13]. Direct contact with paper (predominantly thermal paper), toys, and medical equipment proportionally increase the potential skin exposure to BPA. The third main route of exposure is inhalation of BPA-containing vapors, dust, mists, and gases [3]. Several biomonitoring studies have presented measurable levels of BPA and its metabolites (BPA glucuronide and BPA sulfate) in urine from people in industrialized countries [14,15,16]. Additionally, BPA has been detected in maternal blood plasma, amniotic fluid, and breastmilk [17,18]. Biochemical assays have demonstrated that BPA can act as xenoestrogen and has the ability to interfere with and prevent the binding of natural estrogen hormone to its nuclear receptor (ERα and ERß) [11,19]. On the other hand, BPA can imitate hormone function; therefore, it can magnify the effect of endogenous hormones [20,21]. Moreover, BPA can bind to the membrane G protein-coupled estrogen receptor (GPER, also named GPR30) [22], which can stimulate fast non-genomic estrogenic responses [8,23]. Additionally, BPA’s chemical properties enable its binding to estrogen-related receptor γ (ERR-γ) [8,22,24,25], androgen receptor (AR) [8,22,24,26], thyroid hormone receptor subtypes (THRα and THRß) [8,22,24], glucocorticoid receptor (GR) [8,24,27], progesterone receptor (PR) [8,24], and peroxisome proliferator-activated receptor γ (PPAR-γ) [8,22,24]. Indeed, it has been previously proposed that BPA, through its binding to GPER and GR, mediates the expression of the receptor for activated C kinase 1 protein (RACK1) [27], a scaffolding protein involved in several signaling pathways, including those related to cancer [28] and immune cell responses [29]. Namely, BPA, among other EDCs, could impact the tumor microenvironment (TME), with protein RACK1 playing a pivotal role due to its proinflammatory effects and its ability to bind key factors necessary for cell proliferation, migration, invasion, metastasization, and epithelial–mesenchymal transition (EMT) [30]. In addition, other extranuclear receptors, such as membrane steroid receptors (mSRs), have been studied for their ability to bind and utilize hormones and hormone-like substances (e.g., BPA), emphasizing their emerging role in mediating rapid non-genomic signaling through the modulation of different intracellular molecular pathways, which could contribute to the development and progression of hormone-related cancers [31]. A well-functioning immune system is crucial for maintaining the integrity of the organism, as outlined in the IPCS/WHO guidance for immunotoxicity risk assessment of chemicals (IPCS/WHO 2012); thus, its impairment may lead to serious health issues [32]. In this context, it was proposed that BPA and other bisphenols can modulate the differentiation and activation of immune cells, confirming their immunotoxic effects [33].

It has been established that BPA can have a negative effect on human health, including increased female and male infertility, precocious puberty, genital tract abnormalities, polycystic ovary syndrome (PCOS), and premature ovarian insufficiency (POI) [11,34,35,36,37,38]. Some studies have reported the negative association between urinary concentrations of BPA and serum anti-Müllerian hormone (AMH) levels, an important marker of ovarian reserves and fertility in women [39]. Not only has BPA shown thyroid disrupting effects, but its harmful impacts on the nervous and immune systems have also been reported [8,40,41]. Several animal experimental studies have also demonstrated that prenatal exposure to EDCs such as BPA is associated with increased risk of obesity, peripheral insulin resistance, and non-alcoholic fatty liver disease [42,43]. Correlations between BPA exposure and hormonal carcinogenesis have been explored in animal studies, where results have pointed to an increased risk of breast, prostate, and uterine cancers in BPA-exposed rats and mice [37,44,45,46].

An important question is whether BPA is also associated with the development of human ovarian cancer (OC), a severe and highly fatal human disease. Ovarian cancer (OC) is one of the most common gynecological cancer diagnoses in women and represents the leading cause of death from gynecologic malignancies worldwide. Incidences of hormonal cancers are continuously rising, including OC, with the highest incidence rate being in Europe and North America. In the majority of cases, patients are diagnosed in advanced stages of disease, with a 5-year survival rate below 45%, mostly due to the detection of disease when already widespread [47,48,49,50]. Ovarian cancers are a heterogeneous group of malignancies and are commonly classified into epithelial ovarian cancers (EOCs), which represent 90% of all cases, and non-epithelial ovarian cancers (NEOCs). Among all EOCs, high-grade serous carcinomas are the most common histological subtype, accounting for around half of all EOCs [51]. The pathophysiology of OC and borderline ovarian tumors (BOTs), which rarely develop into aggressive ovarian cancers, is still a topic of research that, to date, is still not completely understood. Nonetheless, it has been described that genetic, reproductive, hormonal, and behavioral factors could contribute to an increased risk of OC [52]. Additionally, several risk factors for OC have been described, such as nulliparity, menopausal hormone therapy use, a lower prevalence of protective factors (e.g., lactation, oral contraceptive methods), and familial predisposition [47,53]. Namely, more than 20% of OCs are due to mutations in tumor suppressor genes, predominantly *BRCA1* and *BRCA2* [53]. The causes of OC are particularly poorly understood in younger women in their reproductive years.

According to the findings of a case–control study, some lifestyle factors such as the consumption of vegetables, vitamin supplements, and beta-carotene are correlated with a reduced rate of OC, while higher cholesterol intake is associated with an increased risk of OC in women [54]. Exposure to environmental pollutants, such as ionizing radiation, asbestos, pesticides, and industrial effluents, is among the risk factors for OC [55]. In recent years, more attention has been focused on possible toxic effects of BPA on the ovary as a hormone-sensitive organ. The correlation between BPA exposure and ovarian carcinogenesis has been less extensively researched. From the perspective of EOC, the impact of BPA is usually investigated on various epithelial ovarian cancer cell lines under in vitro conditions, and negative effects such as the induction of cell proliferation [56,57], inhibition of apoptosis [58], progression of ovarian cancer metastasis [59], and stimulation of pro-angiogenic activity in epithelial ovarian cancer cells have been demonstrated [60]. Multiple studies on animal models have suggested that prolonged exposure to BPA from early stages of life stimulates changes in the ovaries, including alterations in ER signaling, leading to changes in ovarian morphology and ovulation [61,62]. Furthermore, PCR analysis has shown that exposure to BPA modifies cell cycle genetically programmed processes, such as the upregulation of mRNAs (e.g., CDK4, cyclin D1, cyclin A, proliferating cell nuclear antigen, E2F transcription factor (E2F)3 and E2F1) and downregulation of mRNAs (e.g., p21, GADD45, Weel-1), both associated with cell proliferation [62,63]. It is of great interest that some studies have confirmed the crucial role of BPA in the pathogenesis of ovarian cancer by inducing cell signaling pathways (e.g., MAPK/ERK and PI3K/AKT pathways) [64]. Prenatal exposure to BPA has been associated with the inhibition of apoptotic gene expression (e.g., *CAD*, *FAS*, *RAIDD*, *FADD*, *BOK*, and *caspase*), induction of pro-survival gene expression (e.g., *Mcl-1* and *Bcl-x1*), and stimulation of ovarian cancer cell growth through the specific signaling axis ER-CXCL12-CXCR-4 [62,65]. As already mentioned, OC is often diagnosed in advanced stages, with present metastasis in the peritoneal cavity [66]. It has been stated that exposure of granulosa lutein cells in the ovaries to BPA induces the expression of matrix metalloproteinases (MMPs), such as MMP-9, which alters the extracellular matrix in the basal membrane, promoting metaplastic transformation of normal ovarian cells and, therefore, tumor invasion [61,67,68]. Several studies have investigated unfavorable BPA effects on different human organs through the stimulation of reactive oxygen species (ROS) production. Additionally, research on ovarian cancer stem cells in mice has shown that BPA administration induces ROS production, which decreases the expression of the antioxidant genes *Superoxide dismuatase 1* (*SOD1*), *Superoxide dismuatase 2* (*SOD2*), *Catalase* (*CAT*), *Glutathion peroxidase* (*GPX1*), and *Forkhead box O3* (*FOXO3*), leading to BPA-induced oxidative stress [69]. Another study on a mouse model using a PCR technique demonstrated reduced expression of anti-inflammatory genes (*IFN-γ*, *TNF-α*, *TGF-ß*, and *IL-6*) when exposed to BPA, while the expression of nuclear factor NF-kB, responsible for cellular responses to different stimuli in various epithelial ovarian cells, was increased, which contributed to the inflammatory response in the ovaries with induced mortality in mice [70].

To our knowledge, the content of BPA in biological samples from women with EOC or epithelial borderline ovarian tumors (EBOTs) has not yet been investigated. Therefore, the purpose of this study was to determine the concentrations of BPA in the urine of young, reproductive-age, and older women with EOC or EBOT and find their possible associations with kidney function at the molecular level, urine and blood biochemical parameters related to metabolism, AMH as a marker of ovarian reserves, and lifestyle habits in these women compared to healthy controls.

## 2. Results

### 2.1. Characteristics of the Study Population Compared to Healthy Controls

The basic characteristics of the EOC/EBOT group of women (*n* = 24) and healthy controls (*n* = 26) are presented in Table 1. In the control group, represented by healthy young women without ovarian malignancies, the ages were distributed between 23 and 39 years, with a median age of 34 years. Within EOC/EBOT group 1 (*n* = 13), represented by women of reproductive age with EOCs or epithelial borderline ovarian tumors (EBOTs), the ages were distributed between 16 and 49 years, with a median age of 36 years. Meanwhile, in EOC/EBOT group 2 (*n* = 11), represented by women in the post-reproductive period with EOCs or EBOTs, the ages were distributed between 50 and 72 years with a median age of 61 years.

Women in EOC/EBOT group 2 were significantly older compared to women in the control group (*p* < 0.001) or women in EOC/EBOT group 1 (*p* < 0.001). There was no significant difference in age between the control group and EOC/EBOT group 1 (*p* = 0.921). Women in EOC/EBOT group 1 were significantly younger than women in EOC/EBOT group 2 (*p* < 0.001).

The median body mass index (BMI) of the control group (24.5 kg/m^2^) was comparable to the median BMI of EOC/EBOT group 1 (24 kg/m^2^), with both values in the normal range (18.5–24.9 kg/m^2^) for adults (Table 1). However, the median BMI for EOC/EBOT group 2 (30.5 kg/m^2^) was higher and in the obesity range (30–34.9 kg/m^2^) for adults. Namely, women in EOC/EBOT group 2 had a significantly higher BMI compared to women in the control group (*p* = 0.002) and women in EOC/EBOT group 1 (*p* = 0.003). In contrast, there was no significant difference in BMI between the control group and EOC/EBOT group 1 (*p* = 1.000).

Four women (4/22:18.2%) from the EOC/EBOT group reported previous ovarian problems, which was comparable to the controls (4/26: 15.4%). The predominant ovarian abnormality in the history of both groups of women was polycystic ovaries (three women in the EOC/EBOT groups and four controls). The difference in the proportions of nulliparous women (without childbirth in the past) between the control and EOC/EBOT groups (8% vs. 36%) was statistically significant, as indicated by both the chi-square test (*p* = 0.0148) and Fisher’s exact test (*p* = 0.0293). This suggests that EOCs and EBOTs were associated with fewer child births in women.

### 2.2. Urine Levels of BPA in Patients with Epithelial Ovarian Cancer and Epithelial Borderline Ovarian Tumors Compared to Healthy Controls

The results of total urinary BPA levels in the control group and EOC/EBOT groups 1 and 2 are presented in Table 2. The LOQ (limit of quantification) for BPA was determined at 0.10 µg/L. BPA was detected above this value in 73% of women in the control group, compared to 92% and 100% in EOC/EBOT groups 1 and 2; nonetheless, the difference was not significant (*p* = 0.097).

Because urine specific gravity (USG) differed notably among all three groups of women, the results for total BPA levels were USG-adjusted.

Women in the control group presented the lowest levels of BPA when expressed per volume or as USG-adjusted BPA levels. The control group was followed by EOC/EBOT group 1, also for USG-adjusted BPA levels. The highest level of BPA was observed in EOC/EBOT group 2 with the corresponding highest USG-adjusted BPA levels, as can be seen in Table 2. Additionally, when groups were compared to each other, there was a statistically significant difference in urinary BPA levels, namely when values were presented per volume (unadjusted) (*p* < 0.001) or were USG-adjusted (*p* < 0.001).

The median BPA levels, both unadjusted and urine specific gravity (USG)-adjusted, were significantly higher in the overall EOC/EBOT group and in the EOC/EBOT 1 subgroup compared to the controls (all *p* < 0.001). These differences remained statistically significant after adjustment for age and BMI (unadjusted BPA: *p* = 0.002 for both comparisons; USG-adjusted BPA: *p* = 0.005 for EOC/EBOT vs. controls, *p* = 0.006 for EOC/EBOT group 1 vs. control group).

### 2.3. Associations Between Urinary BPA and Kidney Function in Women

Urine BPA concentrations in women were associated with urine biochemical parameters such as urine specific gravity (USG), urine creatinine, and the U-NAG/creatinine ratio, which are related to kidney function. To gain a better insight, a multiple linear regression analysis was conducted to assess potential associations between BPA exposure and markers of renal function, including urinary creatinine, the N-acetyl-β-D-glucosaminidase-to-creatinine (NAG/creatinine) ratio, and the estimated glomerular filtration rate (eGFR). Each model included age, body mass index (BMI), pregnancy status (pregnancy “yes” or “no”), and natural log-transformed BPA concentrations analyzed separately as unadjusted or urine specific gravity (USG)-adjusted values as covariates.

#### 2.3.1. Urine Specific Gravity

**Figure 1:**
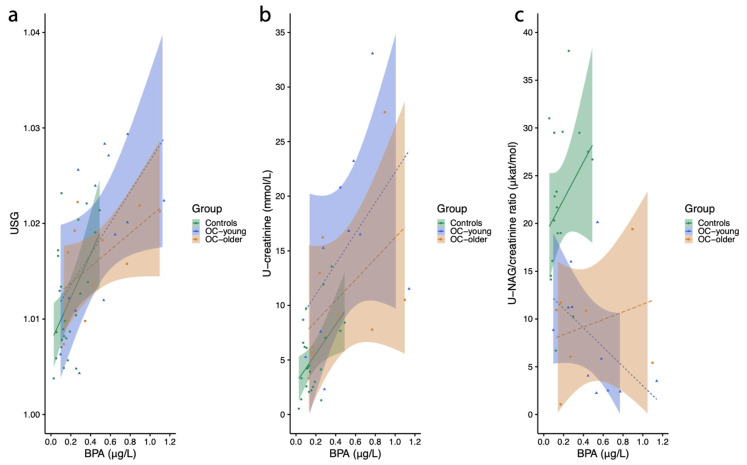
Urine biochemical parameters related to kidney function according to total urinary BPA levels (µg/L) in women in the control group (controls), EOC/EBOT group 1 (OC-young), and EOC/EBOT group 2 (OC-older). (**a**) Urine specific gravity (USG), (**b**) U-creatinine (mmol/L), and (**c**) the U-NAG/creatinine ratio (μkat/mol) (Shapiro–Wilk test followed by Mann–Whitney test, *p* < 0.05). The multiple linear regression analysis of the association between BPA and urinary creatinine or the NAG/creatinine ratio, adjusted for age, BMI, and pregnancy status, showed that log-transformed unadjusted BPA values were significantly associated with urinary creatinine only.

In EOC/EBOT group 1, a statistically significant positive association was observed between urine specific gravity (USG) and urinary BPA levels (r = 0.580, *p* = 0.038) and a marginally positive association in the control group (r = 0.359, *p* = 0.078), while there was no significant association in EOC/EBOT group 2 (r = 0.521, *p* = 0.101) (Figure 1a). The female reference value for USG is wide, between 1.005 and 1.040. All results for women in the control group and both EOC/EBOT groups were in this range. The geometric means were as follows: 1.012 (min. 1.004–max. 1.023) in the control group, 1.018 (min. 1.004–max. 1.029) in EOC/EBOT group 1, and 1.016 (min. 1.007–max 1.022) in EOC/EBOT group 2.

#### 2.3.2. Urinary Creatinine Concentration

Urinary creatinine, which is produced during muscle metabolism, is an important indicator of kidney health. Measuring creatinine in the urine provides valuable information about how the kidneys are working. Elevated creatinine levels in urine can indicate serious health problems, including chronic kidney disease or acute kidney injury.

In this study, a higher level of urinary BPA was significantly positively associated with urinary creatinine concentration in EOC/EBOT group 2 (r = 0.683, *p* = 0.042), while there was only a marginal association in EOC/EBOT group 1 (r = 0.545, *p* = 0.083) and no association observed in the control group (r = 0.307, *p* = 0.145) (Figure 1b).

Multiple linear regression was conducted to examine the association between the following predictors: age, BMI, pregnancy status, log-transformed unadjusted BPA levels (lnBPA), and urinary creatinine in women. The overall model was statistically significant (F(4, 39) = 8.32, *p* < 0.001), explaining 40.5% of the variance in creatinine levels (R^2^ = 0.405). Among the predictors, lnBPA was a statistically significant predictor (β = 0.427, *p* = 0.007), indicating that higher BPA exposure was associated with increased urinary creatinine concentration. Pregnancy status (β = −0.434, *p* =0.016) was a significant predictor and was associated with a decrease of 0.434 standard deviations in urinary creatinine levels, while age (β = −0.259, *p* = 0.10) and BMI (β= −0.037, *p* = 0.78) were not significantly related to urinary creatinine in women. Log-transformed unadjusted BPA concentrations were positively associated with urinary creatinine in the whole EOC/EBOT group of women (*p* = 0.007).

When respecting the USG-adjusted values of BPA, the overall model was statistically significant (F(4, 39) = 5.437, *p* = 0.001), explaining 29.2% of the variance in urinary creatinine levels (R^2^ = 0.292). Among the predictors, lnBPA was not a statistically significant predictor, indicating that USG-adjusted BPA exposure was not associated with urinary creatinine concentration in women. Pregnancy status (β = −0.801, *p* < 0.001) and age (β = −0.365, *p* = 0.040) were significant predictors of urine creatinine in women.

The female reference value for urinary creatinine is in the range of 3.9 and 9.4 mmol/L. The urinary creatinine values in women included in this study were between 0.5 and 46.8 mmo/L, thus indicating lower and higher values compared to reference values. The geometric means were as follows: 13.771 (min. 2.3–max. 46.8) mmol/L in EOC/EBOT group 1, 9.259 (min. 3.3–max. 27.7) mmol/L in EOC/EBOT group 2, and 4.317 (min. 0.5–max. 14) in the control group. We can see that the geometric mean values for urinary creatinine were higher in EOC/EBOT group 1 women compared to the reference value. This may reflect cancer or even higher BPA exposure.

#### 2.3.3. NAG (N-acetyl-beta-d-glucosamidase)/Creatinine Ratio

Urinary *NAG* is a marker of renal tubular damage, and it is widely used as a valuable biomarker in both acute and chronic kidney disease. The NAG/creatinine ratio is an independent predictor for advanced diabetic kidney disease. We analyzed the correlation between NAG (N-acetyl-beta-d-glucosamidase/creatinine ratio) in the urine and urinary level of BPA. In EOC/EBOT group 1, there was an inversed association between the urinary NAG/creatinine ratio and urinary BPA level (r = −0.615, *p* = 0.033). In contrast, there were no significant associations in the control group (r = 0.158, *p* = 0.519) or EOC/EBOT group 2 (r = 0.036, *p* = 0.939) (Figure 1c).

The multiple linear regression analysis showed that the model predicting the NAG/creatinine ratio from the unadjusted BPA, age, BMI, and pregnancy status did not reach statistical significance (F(4, 33) = 1.428, *p* = 0.247), suggesting that these predictors collectively did not account for a significant proportion of variance in the NAG/creatinine ratio. Similarly, when respecting the USG-adjusted values of BPA, the overall model was not statistically significant (F(4, 33) = 1.403, *p* = 0.255), indicating that these predictors did not explain significant variance in the NAG/creatinine ratio. This indicates that urinary BPA is not associated with the NAG/creatinine ratio in women.

The reference value of the NAG/creatinine ratio is 1.45–9.19 qkat/mol. In EOC/EBOT group 1, the geometric mean was 7.624 (min. 2.26–max. 94.3) *qkat/mol*, while in EOC/EBOT group 2, the geometric mean was 7.148 (min. 1.07–max. 19.40) *qkat/mol*, and in the control group, it was 20.684 (min. 6.7–max. 48.08) qkat/mol. The control group had a urinary NAG/creatinine ratio higher than the reference range, indicating transient kidney injury due to late pregnancy. In EOC/EBOT group 1, there were seven women who expressed a very high NAG/creatinine ratio, which could indicate early kidney injury in spite of their young reproductive age, possibly due cancer.

#### 2.3.4. Glomerular Filtration Rate (eGFR) Estimation

The kidneys filter blood by removing waste and extra water to make urine. The glomerular filtration rate (GFR) shows how well the kidneys are filtering the blood. In this study, the Modification of Diet in Renal Disease (MDRD) formula was used for GFR estimation (eGFR). The results are presented as the mean value, standard deviation, and *p*-value. The equation includes parameters such as sex, age, race, and serum creatinine levels. The results showed that women in the control group had statistically significantly higher levels of the eGFR compared to women in EOC/EBOT groups 1 and 2 (139.69 +/− 29.52 vs. 91.17 +/− 21.29; *p* < 0.05). Women in the control group had significantly higher levels of the eGFR compared to those in EOC/EBOT group 1 (139.69 +/− 29.52 vs. 100.85 +/− 19.11; *p* < 0.05) or EOC/EBOT group 2 (139.69 +/− 29.52 vs. 78.6 +/− 19.89; *p* < 0.05). The lowest levels of the eGFR were presented in EOC/EBOT group 2 and were significantly lower than those measured in EOC/EBOT group 1 (78.6 +/− 19.89 vs. 100.85 +/− 19.11; *p* < 0.05).

The estimated GFR, calculated using the MDRD formula, was in the normal range (≥90 mL/min/1.73 m^2^) in all women in the control group (100%; 26/26). Among EOC/EBOT groups, there were four women in group 1 (31%; 4/13) and eight women in group 2 (80%; 8/10) with a reduced eGFR (<90 mL/min/1.73 m^2^). The chi-square test revealed a significantly higher percentage of women with a reduced eGFR in both EOC/EBOT groups 1 and 2 compared to the control group (0% vs. 52.2%; *p* < 0.01). A negative association between urinary BPA levels and the eGFR was observed among all women using the Spearman (r = −0.41; *p* = 0.004) or Pearson (r = −0.43; *p* = 0.002) correlation tests. Later results showed that higher urinary BPA levels were correlated with a reduced estimated GFR and, consequently, damaged kidney function.

A multiple linear regression analysis was conducted to examine whether unadjusted lnBPA, BMI, age, and pregnancy status were associated with the estimated eGFR. The overall model was statistically significant (F(4, 42) = 6.28, *p* < 0.001), indicating that the predictors collectively explained 31.5% of the variance in the eGFR (R^2^ = 0.315). Among the predictors, the unadjusted lnBPA was not a significant predictor (*p* = 0.324), indicating that BPA exposure was not associated with the eGFR. Age was the only significant predictor; higher age was associated with a lower eGFR in women. When a similar analysis was performed using the USG-adjusted BPA values, the overall model was also statistically significant (F(4, 42) = 6.979, *p* < 0.001), indicating that predictors collectively explained 34.2% of the variance in the eGFR (R^2^ = 0.342). Among the predictors, USG-adjusted lnBPA was not statistically significant (β = −0.255, *p* = 0.103), indicating that BPA exposure was not associated with the eGFR in women. Age (b = −0.593, *p* < 0.001) was the only significant predictor; higher age was associated with a lower eGFR in women.

### 2.4. Associations Between Urinary BPA, Platelets, and Triglycerides in Blood of Women

The aim of this study was to identify whether urine concentrations of BPA were also associated with some blood biochemical parameters related to the formation of clots that stop or prevent bleeding and the metabolism of lipids.

#### 2.4.1. Mean Platelet Volume

**Figure 2:**
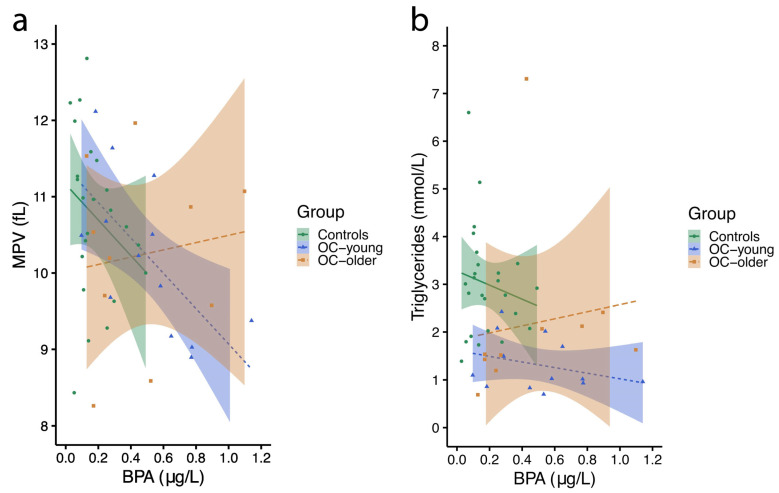
Blood biochemical parameters related to total urinary BPA levels (µg/L) for control group (controls), EOC/EBOT group 1 (OC-young), and EOC/EBOT group 2 (OC-older) women. (**a**) Triglycerides (mmol/L) and (**b**) the mean platelet volume (MPV) (fL) (Shapiro–Wilk test followed by Mann–Whitney test, *p* < 0.05). However, the multiple linear regression analysis of the association between BPA and MPV or triglycerides, adjusted for age, BMI, and pregnancy status, showed that log-transformed unadjusted or USG-adjusted BPA concentrations were not significantly associated with MPV or blood triglycerides in women.

Platelets (also called thrombocytes) are small blood cells that stick together to make blood clots that stop or slow bleeding in the case of a cut or injury. Platelets are formed in the bone marrow. The mean platelet volume (MPV) test measures the mean (or average) size of platelets in blood. Looking at the size of platelets provides information about how well they are working. This test can help to diagnose bleeding disorders as well as diseases of the bone marrow. In this study, there was a significant negative correlation between MPV and the urinary BPA level in EOC/EBOT group 1 (r = −0.726, *p* = 0.005), with a tendency toward a negative association in the control group (r = −0.354, *p* = 0.090) and no association in EOC/EBOT group 2 (r = −0.006, *p* = 0.987) (Figure 2a).

Multiple linear regression was conducted to evaluate whether unadjusted BPA, BMI, age, and pregnancy status predicted MPV in women. The overall model was not statistically significant (F(4, 42) = 1.76, *p* = 0.154), suggesting that the included predictors did not account for a significant amount of variance in MPV. Similar observations were made regarding the USG-adjusted BPA values (F(4, 42) = 0.98, *p* = 0.431). This suggests that urinary BPA is not associated with MPV in women.

The female reference value of MPV is 9.6–12 fL. The geometric mean value was 10.177 (min. 8.9–max. 12.1) fL in EOC/EBOT group 1 women, 10.174 (min. 8.3–max. 12) fL in EOC/EBOT group 2 women, and 10.804 (min. 8.4–max. 14.2) fL in the control group. The mean values were quite comparable and within the reference value range. In both EOC/EBOT groups 1 and group 2, a small number of women (one and two women, respectively) had lower values, while in the control group of women, there were four women with higher MPV values, possibly due to late pregnancy.

#### 2.4.2. Blood Triglycerides

Triglycerides are a type of fat (lipid) found in the blood. When eating, the body converts any calories it does not need to use right away into triglycerides. The triglycerides are stored in the fat cells. High triglycerides may contribute to hardening of the arteries or thickening of the artery walls (arteriosclerosis), which increases the risk of stroke, heart attack, and heart disease. Extremely high triglyceride levels can also cause acute inflammation of the pancreas (pancreatitis). Regarding lipid profile measurements, we observed a positive association between urinary BPA levels and blood (serum) levels of triglycerides in EOC/EBOT group 2 (r = 0.756, *p* = 0.011), with no significant association observed in the control group (r = −0.056, *p* = 0.791) or EOC/EBOT group 1 (r = −0.232, *p* = 0.445) (Figure 2b).

Multiple linear regression was conducted to examine whether unadjusted lnBPA, BMI, age, and pregnancy status were associated with serum triglycerides. The overall model was statistically significant (F(4, 43) = 3.876, *p* = 0.009), indicating that the predictors collectively explained 19.7% of the variance in blood triglycerides (R^2^ = 0.197). When a similar analysis was performed using the USG-adjusted BPA values, the overall model was statistically significant (F(4, 43) = 3.920, *p* = 0.008), indicating that the predictors collectively explained 19.9% of the variance in blood triglycerides (R^2^ = 0.199). Among the predictors, USG-adjusted BPA was not a statistically significant predictor (b = −0.063, *p* = 0.713), indicating that BPA exposure is not associated with blood triglycerides. Pregnancy status (b = 0.545, *p* = 0.008) was the only significant predictor; therefore, it can be posited that pregnancy is associated with higher blood triglycerides.

The recommended reference value is below 1.7 mmol/L. In EOC/EBOT group 1 women, the geometric mean value was 1.217 (min. 0.7–max. 2.4) mmol/L; in EOC/EBOT group 2 women, it was 1.784 (min. 0.7–max. 7.3); and in the control group, it was 2.801 (min. 1.4–max. 6.6) mmol/L. In EOC/EBOT groups 1 and 2, there were 3 (23%) and 4 (36%) women, respectively, with values higher than recommended; in the control group, 24 (92%) women exceeded the reference values, possibly due to late pregnancy.

There was no significant association between urine BPA and several other biochemical parameters in the urine and blood of women, including those related to liver function.

### 2.5. Serum AMH and Urinary BPA Levels in Women

The geometric mean concentrations of serum AMH were 0.85 (min. 0.33 and max. 2.74) ng/mL in the controls and 0.58 (min. 0.04 and max. 4.19) ng/mL in EOC/EBOT group women (group 1: 0.85, min. 0.25 and max. 4.19; group 2: 0.08, min. 0.04 and max. 0.17).

There was a negative correlation between serum AMH levels and female age in the whole cohort of women (r = −0.5547, *p* = 0.0022). This was also observed in the EOC/EBOT group of women, who consisted of young (group 1) and older women (group 2) (r = −0.9205, *p* = 0.0004), but not in young controls, who were in a reproductive period of life (r = −0.2744, *p* = 0.2555).

In the whole cohort of women, there was no association between unadjusted urinary BPA and serum AMH levels (r = −0.1235, *p* = 0.5393). We did not observe any association between urinary BPA and serum AMH levels either in the controls (r = −0.2314, *p* = 0.3556) or in women from the EOC/EBOT group (r = 0.2769, *p* = 0.4708). Similarly, we did not observe any associations between urinary levels of BPA adjusted for USG (r = 0.04852, *p* = 0.8219) with serum AMH levels in all women.

### 2.6. Lifestyle Habits in Women with Ovarian Cancer Compared to Controls

A comparison of lifestyle habits, determined using a validated questionnaire, between the control group of young healthy women without ovarian malignancies and the EOC/EBOT group of women with histopathologically confirmed EOC or EBOT is given in Table 3.

Women in the EOC/EBOT group were significantly older than women in the control group (*p* < 0.001). There were no marked differences in the age of menarche, length of menstrual cycle, or educational level between the groups. However, there was a notably higher percentage of smokers in the control group compared to those in the EOC/EBOT group (Table 3). While there were similar coffee drinking habits between the control and study groups, with the majority of them being coffee consumers, significantly more women in the EOC/EBOT group consumed a higher number of coffees per day—three, four, or ten cups of coffee per day (*p* = 0.016). Moreover, remarkably more women in the EOC/EBOT group consumed folic acid during their pregnancy compared to women in the control group (*p* < 0.001). On the other hand, there was significantly lower consumption of dietary supplements among women in the EOC/EBOT group (*p* = 0.015), as can be seen in Table 3.

The majority of women in the control group did not use water purification systems (86.4%), while there were significantly more women in the EOC/EBOT group who used a water filter or water softener (*p* < 0.001) (Table 3). A higher percentage of women in the EOC/EBOT group had consumed food from PVC containers or PVC bags/film within 24 h or 2 days of urine sampling compared to healthy controls (*p* = 0.004 and *p* = 0.012, respectively). The majority of participants in both groups reheated food in plastic containers less than once per week. About half of the participants in both groups had the habit of refilling single-use plastic bottles. A sport bottle was more often last used within 24 h or 2 days of urine sampling in the EOC/EBOT group (*p* = 0.004), while the majority of women in the control group last used a sport bottle more than 2 days before urine sampling (Table 3).

### 2.7. Lifestyle Habits of Women Associated with BPA Concentrations in Their Urine

Correlations between lifestyle habits and urine BPA concentrations, as determined by analysis of variance (one-way ANOVA or Kruskal–Wallis test), are presented in Table 4. The analysis was performed on women in the control group, women in the EOC/EBOT group with histopathologically confirmed epithelial ovarian cancer (EOC) or epithelial borderline ovarian tumors (EBOTs), and the whole cohort of women (the control and EOC/EBOT groups together). Several associations of urinary BPA with lifestyle habits were observed in the overall population of women, with only a few in the control group. However, there were no associations in the EOC/EBOT group of women, most likely due to a relatively low number of women being included in this group.

In Table 4, we can see all those lifestyle habits that were statistically significantly associated with urinary BPA in women, including intake of dietary supplements, the use of plastic material for storing foods and drinks, and having amalgam fillings. In all women, intake of dietary supplements was associated with significantly lower urinary BPA levels (*p* = 0.032), while no such association was observed in the control group (*p* = 0.111), as seen in Table 4. A positive association between the habit of refilling single-use plastic bottles and BPA levels was observed for the whole cohort of women (*p* = 0.048), with a higher tendency observed in the control group (*p* = 0.051). Among the whole cohort of women, the level of urinary BPA was negatively associated with the time interval between the last intake of food from PVC containers and urine sampling (*p* = 0.023), but this was not the case in the control group (*p* = 0.166) (Table 4). The number of amalgam dental fillings was significantly associated with higher urinary BPA levels in women in the control group (*p* = 0.035) but not in other women. It is important to emphasize that healthy controls differed from the EOC/EBOT group of women precisely with regard to the use of dietary supplements and the use of plastic products for food and beverages, which also appears to be associated with urinary BPA concentrations in women. On the other hand, there was no significant association between urinary BPA, smoking, coffee consumption, and the use of a water purification system, lifestyle habits that also differed between the controls and EOC/EBOT group of women.

## 3. Discussion

This study presents data on BPA exposure in women with EOC or EBOT compared to healthy controls. The results of this study suggest that unadjusted or USG-adjusted BPA levels could be increased in women with EOC or EBOT compared to healthy controls and positively associated with urine creatinine and potential impaired kidney function. Additionally, potential sources of BPA exposure were identified among selected groups using a validated questionnaire, with women in the EOC/EBOT group being more exposed to different plastic products. Moreover, the study indicated that some lifestyle habits, including the habit of refilling used plastic bottles, could correlate with higher urinary BPA levels in the whole cohort of women.

To the best of our knowledge, this paper is the first to study BPA levels in the urine of women with ovarian malignancies and their correlation with kidney function. To date, there have been several reports about urinary BPA measurements in general populations around the world [71,72,73,74]. The geometric means of urinary measurements of BPA in our study were below the latest human biomonitoring guidance values (HBM-GVs) for adults (230 µg/L) derived by the European Humam Biomonitoring Initiative (HBM4EU) based on data from different European countries [75]. Nonetheless, our results also presented lower levels of urinary BPA for women in comparison to biomonitoring data in Slovenia for women of childbearing age in the general population published 6 years ago [6]. Data from non-European countries showed higher BPA levels for adult females in U.S. and Korean populations, respectively [76].

In spite of the relatively low exposure of women in Slovenia to BPA in comparison to other countries, our observation showed that the content of unadjusted BPA in the urine of women with EOC or EBOT was positively associated with urinary creatinine after age, BMI, and pregnancy adjustment using a multiple linear regression analysis. This suggests potential kidney injury due to an elevated urinary creatinine level. This may be in accordance with the observation that geometric mean values for urinary creatinine were higher in EOC/EBOT group 1 women compared to the reference value. This may reflect the cancer disease or even higher BPA exposure according to the multiple linear regression analysis. We also found some other associations between urinary BPA and kidney parameters such as the NAG/creatinine ratio, but they were not significant after adjustment for age, BMI, and pregnancy.

To our knowledge, no data have been published yet about BPA’s impact on kidney function in women with ovarian malignancies. Nonetheless, it has been proposed that urinary excretion of any biomarker that is filtered by the glomerulus, including creatinine, is affected by the glomerular filtration rate (eGFR) and amount of water in the urine [77]. There is some evidence that urinary NAG excretion is correlated with several complications of chronic diseases such as type 2 diabetes and atherosclerosis, including neuropathy, retinopathy, and nephropathy, which is caused by proximal tubular cell injury [78,79]. Although there was no significant association between urinary BPA and the NAG/creatinine ratio in women after adjustment for age, BMI, and pregnancy, there was a significantly inversed correlation between BPA and creatinine in the urine of younger women with EOC or EBOT. This could be explained by the fact that younger women had preserved kidney function with a better eGFR compared to older women with malignancies, which means that higher BPA clearance correlated with a lower NAG/creatinine ratio.

Our data showed that the eGFR was reduced in 80% of older and 31% of younger women with EOC or EBOT but in none of controls. The kidney function of women with ovarian malignancies was impaired in accordance with the BPA level in their urine, with the eGFR being under the normal level (90 mL/kg/m^2^) even though most of the recommendations mention a fixed cut-off value at 60 mL/kg/m^2^ [80,81]. Like in our study, some data in the literature support the role of BPA in the pathogenesis and progression of kidney disease and emphasize an inverse relationship between plasma BPA and renal function expressed by the eGFR [82]. Moreover, there have been several studies focusing on the relationship between urinary BPA and the eGFR with reports of their negative relationship [83,84,85,86], with only one publication showing an inverse correlation [87]. This could result from a loss of kidney function and a lower eGFR, leading to decreased BPA excretion.

Although BPA has been well studied regarding its toxicological properties, less is known about its effect on blood components, such as platelets, which are crucial for homeostasis and thrombus formation [88]. Our observations of the negative correlation between urinary BPA and MPV in younger women with EOC or EOBT could be explained by BPA-induced inflammation since low MPV has been linked to high inflammatory diseases [89]. However, no significant association was found between urinary BPA and blood MPV or triglycerides after adjustment for age, BMI, and pregnancy using a multiple linear regression analysis.

Our data showed no associations between urinary concentrations of BPA and serum AMH as an indicator of ovarian reserves in women, like in some other studies [90]. Oppositely, in some other studies, it was found that urinary BPA concentrations were negatively associated with serum AMH levels in women who were mostly attending fertility clinics because of their problems with conception [39,91]. However, we studied a different population of women with EOC and EBOT in whom the mechanism of BPA influence may be different, and it is also not known how the disease itself and subsequent treatment may affect ovarian reserves in these women.

An analysis of lifestyle habits studied using a validated questionnaire showed some important differences between women with EOC or EBOT and healthy controls. Our observations showed that women with ovarian malignancies consumed fewer dietary supplements than healthy controls, which could be the consequence of a gradual increase in the use of different dietary supplements among young healthy adults [92]. Furthermore, we would like to reveal the results on plastics. Women with ovarian malignancies used water purification systems (e.g., water filters or a water softener) in a significantly higher ratio than healthy controls. Additionally, they more frequently consumed food from PVC containers or PVC bags/film within 24 h or 2 days of urine sampling compared to the controls. The pathogenic role of PVC exposure in carcinogenicity in humans has already been established [93]; however, there is only some evidence for hepatocellular carcinoma and angiosarcoma [94] and not for ovarian cancer.

Lastly, we found some associations between some lifestyle habits and urinary BPA levels in the women included into this study. Our results indicate that intake of dietary supplements was inversely correlated with BPA urinary levels in the entire cohort of women. Indeed, there have been predictions, based on in vitro studies, that some dietary supplements, like probiotics, could have the capacity to bind to different pollutants (e.g., BPA) in the gastrointestinal tract and, therefore, prevent BPA absorption and negative effects on human health [95]. Dietary exposure to BPA through the habit of refilling single-use plastic bottles correlated significantly with higher BPA levels in the entire cohort of women and controls. It has been established that bioactive BPA is released from already used new polycarbonate bottles and migrates into the liquid at room temperature [96]. Moreover, exposure to elevated temperatures, for example, sterilization of baby feeding bottles or applying boiling water to drinking bottles, may lead to increased hydrolysis of polycarbonate polymers, which could result in a greater extent of BPA migration [97]. Our observation of the entire cohort of women showed that urinary BPA concentrations were negatively associated with the time interval between the last food intake from PVC containers and urine sampling. Knowing that the majority of all BPA exposure is from dietary sources [98] and that BPA in human adults is rapidly metabolized and cleared out in under 24 h of exposure [2,99], it could be presumed that the more time that passes from BPA exposure to urine sampling, the lower the concentration of BPA in urine will be. Moreover, studies of human pharmacokinetics have reported that urinary BPA levels peak between 1 and 6 h after administration, with the BPA urinary elimination half-life set between 4 and 5.4 h [100,101].

Since EOC is a gynecological malignancy that primarily occurs in postmenopausal women, with only 10 to 15% found in premenopausal women [102,103], during this study, we faced difficulties with recruiting young patients with EOC, a particularly sensitive group of women, as ovarian malignancy occurs in their reproductive period of life and can negatively affect their fertility. A notable limitation to our research was the relatively low number of young women with ovarian malignancies in our study cohort. However, all women with EOC and EBOT were precisely chosen on the basis of their histopathological diagnosis, as only those with epithelial ovarian malignancies were included. In addition, another limitation of our study could be the selected control group represented by pregnant women, as some metabolic changes could be a result of pregnancy itself. Therefore, we performed a multiple linear regression analysis to evaluate associations of urinary BPA with other urine and blood parameters, including pregnancy adjustment. Nonetheless, women in the control group were otherwise healthy and without any history of chronic disease or malignancy.

Because of the short half-life of BPA, spot urinary BPA concentrations reflect exposure within a relatively short period of time before urine collection and may differ from chronic exposure [104], which is an additional limitation to our study. Since there is considerable within-person and between-person variability of BPA concentration in spot urine samples [105,106], due to daily diet changes, better options for the evaluation of BPA exposure have been proposed, such as repeated random urine sample collection [107]. However, women with EOC and EBOT were included in the study at the time of hospital admission with planned surgery for the next day; consequently, there was lack of time for collecting several repeated urine samples.

Furthermore, it should be underlined that ovarian malignant cells modify several metabolic pathways to satisfy their fast proliferation, including the acceleration of glucose, amino acid, and lipid uptake, which could also correlate with an impaired lipid profile [108]. Moreover, there are some data on reduced renal function and the dilatation of the upper urinary tract in patients with ovarian cancer at the time of diagnosis [109], implying a non-negligible limitation to our study.

The results of this study suggest that urinary BPA may be associated with ovarian cancer. Many new questions arise about how elevated BPA levels actually affect the body and metabolism in women with ovarian malignancies. We have studied the effect of BPA on the kidneys and AMH, but many other negative effects are possible; these effects, including oxidative stress and inflammation, need to be researched in the future.

Finaly, for our future direction, we would like to analyze toxic properties of other bisphenol substitutes, such as bisphenols F (BPF), S (BPS), Z (BPZ), and AF (BPAF), that are increasingly replacing BPA with a robust date on their potenital toxicity. Not only these BPA alternatives exhibit endocrine activity, but they might also be even more harmful than BPA. To capture a fuller exposure profile to emerging endocrine-disrupting chemicals, including BPA and its substitutes, a multiplex analysis platform using LC-MS/MS or GC-MS/MS should be considered in future studies.

## 4. Materials and Methods

### 4.1. Study Population

This study was reviewed and approved by the Republic of Slovenia National Medical Ethics Committee with corresponding ethical approval No. 0120–215/2023/6. Written informed consent was obtained from all subjects involved in the study. Participation was voluntary, with each participant having the right to withdraw from the study at any time.

A total of 50 women were included in this cross-sectional, controlled study: (i) 26 in the control group of healthy women (controls) and (ii) 24 in the study group of women with epithelial ovarian cancer (EOC) or epithelial borderline ovarian tumor (EBOT) (EOC/EBOT group). The control group consisted of women aged 23–39 years with singleton pregnancies who were scheduled for a caesarean section at term (37–42 th weeks of gestation) and gave birth between September 2021 and September 2022 at the Department of Obstetrics, Division of Gynaecology and Obstetrics, University Medical Centre Ljubljana. The exclusion criteria for the control group were as follows: (i) current or previous malignant diseases; (ii) chronic diseases (i.e., diabetes mellitus; hypertension; chronic heart disease; cardiovascular disease; chronic lung disease, including asthma; autoimmune disease, including rheumatoid arthritis and systemic lupus erythematosus; chronic kidney disease; inflammatory bowel disease; neurological disorders; osteoporosis; and anxiety and depression disorders); (iii) multiple pregnancies; (iv) premature labor (before the 37th week of gestation); and (v) stillbirth.

The EOC/EBOT group included women who were diagnosed with EOC or EBOT and had scheduled surgery between September 2021 and December 2022 at the Department of Gynaecology, Division of Gynaecology and Obstetrics, University Medical Centre Ljubljana. The EOC/EBOT group was further divided into two groups of women: group 1 was represented by 13 women in the reproductive period of life (aged from 16 to 49 years) and group 2 was represented by 11 women in the post-reproductive period (aged from 50 to 72 years). Only women with histopathologically confirmed EOC or EBOT were included in the study group. The exclusion criteria for the study group were histopathologically confirmed non-epithelial ovarian cancer, non-epithelial borderline ovarian tumors, and benign ovarian changes. None of the women in this group used hormonal contraceptives at the time of diagnosis. Due to ovarian malignancies and their effect on menstrual irregularities, menstrual cycle data may not be reliable.

All participants were informed about the research protocol and aim of the study and received a written explanation about the study. Participants were included in the study only after providing signed informed consent.

### 4.2. Sampling

First-morning urine samples were collected from all participants into 100 mL-volume containers with caps to prevent contamination from plastic packaging. All included women were instructed to correctly collect urine samples into the container. After the assembly of the urine sample into the container, a urine volume of 6 mL was collected with an automatic pipette and squeezed into another 100 mL-volume container; each sample was then directly transferred to the Institute of Clinical Chemistry and Biochemistry (KIKKB), University Medical Centre Ljubljana, for routine diagnostic biochemical analysis. The remaining volume of urine sample was transported to the Clinical Research Centre, University Medical Centre Ljubljana, where all collected urine samples were aliquoted within 2 h after retrieval into 2 mL cryogenic vials, each containing 1.5 mL of urine sample and cryostored. All urine samples were collected and stored in a deep freezer at −80 °C until analysis and were analyzed simultaneously. For the purpose of analysis, all urine samples were shipped frozen on dry ice to the Jožef Stefan Institute (Department of Environmental Sciences) for BPA content analysis.

Concurrently, two venous blood samples were collected: one in a 3 mL collection tube with K2 EDTA for plasma and another in a 6 mL collection tube with no anticoagulant to collect the whole blood for serum processing. After collection, all blood samples were transferred to the Institute of Clinical Chemistry and Biochemistry, University Medical Centre Ljubljana, for routine diagnostic biochemical analyses. All samples were processed under the patient code to protect personal data.

### 4.3. Routine Diagnostic Biochemical Analyses of Biological Samples

Blood samples were analyzed for the following biochemical parameters: kidney function (serum creatinine, glomerular filtration rate (GFR)), liver function (aspartate aminotransferase (AST), alanine aminotransferase (ALT), and gamma-glutamyl transferase (GGT)), and lipid panel (total cholesterol, low-density lipoprotein (LDL) cholesterol, high-density lipoprotein (HDL) cholesterol, and triglycerides). Blood samples were also analyzed for the basic hematological parameters: complete blood count, red blood cell count (RBC), white blood cell count (WBC), platelet count (PLT), hemoglobin (HGB), hematocrit (HCT), mean corpuscular volume (MCV), mean corpuscular hemoglobin (MCH), mean corpuscular hemoglobin concentration (MCHC), red cell distribution width (RDW), and mean platelet volume (MPV).

Urine samples were analyzed for the following biochemical tests: urine specific gravity (USG), IgG and alpha-1-microglobuline (A1M, α1-microglobulin) urinary excretion, urine albumin, urine creatinine, and urinary *N*-Acetyl-ß-glucosaminidase (NAG) levels.

We used the Modification of Diet in Renal Disease (MDRD) formula to calculate the estimated glomerular filtration rate (eGFR). MDRD calculation is based on the following four variables: serum creatinine, age, gender, and ethnicity.

All urine and blood samples were analyzed at the Institute of Clinical Chemistry and Biochemistry, University Medical Centre Ljubljana, with validated laboratory tests used in daily clinal practice. The reference values used in this study were established by this institute and are used in daily clinical practice.

### 4.4. BPA Analysis

Analysis of BPA in urine samples was performed in a qualified laboratory at the Jožef Stefan Institute (Department of Environmental Sciences) based on a previously reported method [6]. Urine samples were deconjugated using ß-glucoronidase/arylsulfatase. Samples were than incubated at 37 °C for 18 h. Subsequently, they were extracted using solid-phase extraction on Oasis HLB cartridges. After elution from polymer sorbent was completed, samples were dried under a gentle stream of nitrogen reconstituted in ethyl acetate (EtAc) and derivatized with *N-tert*-Butyldimethylsilyl-*N*-methyltriflouroacetamide (MTBSTFA). Clean-up was performed using silica columns (500 mg, Isolute Si, Biotage). The samples were later dried under a gentle stream of nitrogen and resuspended in EtAc. Analysis was performed using GC-MS/MS (gas chromatography–tandem mass spectrometry). All BPA measurements are presented in µg/L.

### 4.5. Serum AMH Analysis for Ovarian Reserve Estimation

Serum AMH analysis was performed at the Medicare PLUS Diagnostic Lab in Ljubljana. For the determination of AMH, the electrochemiluminescence immunoassay (ECLIA) method, using the sandwich principle, was employed on a Roche Cobas e411 instrument. Serum samples, controls, and calibrators were warmed to room temperature before analysis. During the first incubation, 50 μL of sample, biotin-labeled AMH-specific monoclonal antibodies, and ruthenium-complex-labeled AMH-specific monoclonal antibodies reacted with each other, and a sandwich complex was formed. During the second incubation, the streptavidin-coated microparticles were added. The formed complex bound to the solid phase due to the interaction between biotin and streptavidin. The reaction mixture traveled to the measurement cell of machine. The microparticles were captured on the electrode surface due to magnetic action. Unbound substances were removed using ProCell M. The voltage at the electrode induced chemiluminescence emission, which was measured using a photomultiplier. The analyzer calculated the concentration using a calibration curve obtained from a two-point calibration. The concentration was expressed as ng/mL.

### 4.6. Questionnaire for the Evaluation of Lifestyle and Habits

On the day of admission to the hospital, a medical doctor interviewed participants and collected data on (a) the region of residency and residential environment, (b) level of education, (c) occupation, (d) nutrition, (e) smoking habits, (f) alcohol and caffeine consumption, (g) female reproduction, and (h) special data on potential sources of BPA exposure using the validated questionnaire. Later included factors were the use of home water filtration systems and/or water softeners; consumption of food from plastic containers, plastic bags, or plastic films; consumption of food reheated in plastic containers; drinking water from plastic bottles and/or water dispensers; habit of refilling single-use plastic bottles; use of plastic sport bottles; and exposure to products potentially containing BPA, such as dental fillings. The questionnaire was previously validated when it was used for national biomonitoring of endocrine-disrupting chemicals in the general Slovenian population [6,110,111].

### 4.7. Study Power Test

Based on the preliminary pilot study, we performed the following power test of our study: https://vbiostatps.app.vumc.org/ps/t-test/ind (accessed on 16 September 2023). BPA concentrations in the urine of women were expressed in μg/L, with values ranging from 0.00 to 1.30 μg/L. In the power test, we entered the standard deviation of the BPA values found in all urine samples (0.2819), the mean value in the subjects (0.4927), the mean value in the healthy controls (0.2233), and the difference between the two mean values (0.2694). When the number of subjects (women) per group was 25, the power of the test (power, computed value) was 1.0 (must be at least 0.8); when the number of subjects was 20, the power of the test was 0.98; and when the number of subjects was 15, the power of the test was 0.93. A test power of 0.8 was achieved by including 11 subjects per group. The numbers of women included in groups of this study were sufficient from the perspective of computed power value.

### 4.8. Statistical Analyses

Statistical analyses were performed using unadjusted or USG-adjusted BPA values. The Shapiro–Wilk test was used as a test of normality to determinate the distribution of the data. If the data were normally distributed, parametric statistical tests were used (Student’s *t*-test to compare the means of two groups; one-way ANOVA test to compare the means of more than two groups); if the data were abnormally distributed, non-parametric statistical tests were used (Mann–Whitney test to compare two sample means; Kruskal–Wallis test to compare more than two sample means; Spearman’s correlation coefficient; Fisher’s exact test for categorical variables).

Since the values were abnormally distributed, the Mann–Whitney test was used to analyze the differences in age and BMI between different groups of women. Comparison of urinary BPA exposure levels between selected groups (control group, study group 1, and study group 2) was performed using the Kruskal–Wallis test. Furthermore, the association between urinary BPA concentration and several biochemical parameters, including AMH, was determined using the Shapiro–Wilk test, followed by the Mann–Whitey test with the use of Spearman’s rank correlation coefficient. Additionally, the Shapiro–Wilk test, followed by Student’s *t*-test, was used to evaluate differences in eGFR calculated using the MDRD formula between groups (control group, study group 1, and study group 2). Spearman’s and Pearson’s rank correlation coefficients were used to determine the association between urinary BPA levels and eGFR. Differences between two groups (control group and study group) in lifestyle habits, which were determined by analyzing selected questionnaire data variables, were calculated with one-way ANOVA or the Kruskal–Wallis test depending on the distribution of data. Additionally, to evaluate the association between urinary BPA concentration and selected questionnaire data variables among groups, one-way ANOVA or the Kruskal–Wallis test were used, depending on the distribution data. Statistical significance was set at *p* < 0.05.

Multiple (multivariable) linear regression analysis was conducted to assess potential associations between BPA exposure and markers of renal function, including urinary creatinine, N-acetyl-β-D-glucosaminidase-to-creatinine ratio (NAG/creatinine), estimated glomerular filtration rate (eGFR), mean platelet volume (MPV), and serum triglyceride concentration (TGC). Each model included age, body mass index (BMI), pregnancy status (yes or no), and natural log-transformed BPA concentrations (analyzed separately as unadjusted or urine-specific-gravity-adjusted) as covariates.

Associations between ovarian cancer (OC; primary outcome) and ln-transformed BPA concentrations (primary exposure) were evaluated using logistic regression models, with pregnant women included as the reference (control) group. Separate models were constructed for unadjusted and urine-specific-gravity-adjusted BPA concentrations. All models were adjusted for age and BMI, risk factors for OC, with mutual correlations below 0.8 to limit multicollinearity.

All statistical analyses were performed using SPSS software (version 29; IBM Corp., Chicago, IL, USA).

## 5. Conclusions

Ovarian cancer is a serious disease that greatly affects a woman’s quality of life and fertility. Our findings show increased urinary BPA levels expressed per volume (unadjusted) or adjusted to urine specific gravity in women with EOC or EBOT compared to healthy controls. Some significant positive associations of urinary BPA concentrations with impaired kidney function, such as increased urinary creatinine, were found in women with EOC and EBOT, which may be related to health problems. Our results show higher levels of BPA in the urine of women with ovarian malignancies. Ovarian cancer is one of the diseases that most severely affects the female (in)fertility and reproductive function, either due to the disease itself or due to the therapy. In the case of hormone disruptors that are associated with the manifestation of ovarian cancer, we can speak of extreme reproductive toxicity.

Moreover, we found some correlations between the urinary levels of BPA and lifestyle habits of women, such as the use of plastic products for storing foods or drinks and the refilling of plastic bottles, using a validated questionnaire. On the other hand, we found no association between urinary BPA concentrations and serum AMH levels as an indicator of ovarian reserves in women. The results of this study are interesting overall and suggest the importance of considering BPA and other EDCs in the management of women with ovarian malignancies, but further research on a larger cohort of women is required to reach definitive conclusions.

## Figures and Tables

**Table 1:** Basic characteristics of 50 women: 24 women in the EOC/EBOT groups and 26 healthy controls.

	Category	Control Group * (*n* = 26)	EOC/EBOT Group 1 ** (*n* = 13)	EOC/EBOT Group 2 *** (*n* = 11)	*p*-Value
N		26	13	11	
Age (years)	Median	34	36	61	*p* < 0.001 (study group 2 vs. control group or study group 1)
	Min–Max	23–39	16–49	50–72	
Body mass index (kg/m^2^)	Median	24.5	24	30.5	*p* = 0.002 (study group 2 vs. control group) *p* = 0.003 (study group 2 vs. study group 1)
	Min–Max	19.3–35.4	18.6–35.9	24.0–38.9	

Legend: * Control group: healthy young women without ovarian malignancies. ** EOC/EBOT group 1: women of reproductive age with histopathologically confirmed epithelial ovarian cancer (EOC) or epithelial borderline ovarian tumor (EBOT). *** EOC/EBOT group 2: women in the post-reproductive period with histopathologically confirmed epithelial ovarian cancer (EOC) or epithelial borderline ovarian tumor (EBOT).

**Table 2:** Descriptive statistics. The results of total urinary BPA levels in the control group and EOC/EBOT groups 1 and 2, demonstrated as µg/L urine and USG-adjusted BPA (µg/L urine) with statistically significant differences for concentrations expressed as µg/L or USG-adjusted values.

Population	N	≥0.10 (µg/L)	Min–Max	GM	95% CI
Total BPA (µg/L)					
Control group	26	77 ^a^	0.10–0.49	0.14	0.11–0.19
EOC/EBOT group 1	13	92 ^b^	0.10–1.14	0.42	0.28–0.63
EOC/EBOT group 2	11	100 ^c^	0.13–1.09	0.36	0.22–0.59
Population	N	≥0.10 (µg/L)	Min–max	GM	95% CI
USG-adjusted BPA (µg/L)					
Control group	26	77 ^a^	0.10–0.61	0.17	0.13–0.22
EOC/EBOT group 1	13	92 ^b^	0.19–1.28	0.47	0.34–0.65
EOC/EBOT group 2	11	100 ^c^	0.16–0.84	0.38	0.26–0.57

^a,b,c^—statistically significant differences (*p* < 0.05). Legend: min.—minimum; max.—maximum; GM—geometric mean; 95% CI—95% confidence interval; BPA—bisphenol A; USG-adjusted BPA—urine-specific-gravity-adjusted BPA.

**Table 3:** Significant differences in lifestyle habits between healthy control group and EOC/EBOT group of women with epithelial ovarian cancer (EOC) or borderline ovarian tumors (EBOTs) regarding answers from validated questionnaire. *p*-value—probability value.

	Category	Healthy Control Group of Women	EOC/EBOT Group of Women	*p*-Value
Age (years)	Median	32.5	42.3	<0.001
Smoking (%)	Yes	24.2	4.8	0.043
	No	75.8	95.2	
	No	19.7	28.6	
Cups of coffee per day (%)	1	36.2	25.0	0.016
	1.5	19.1	12.5	
	2	31.9	25.0	
	2.5	4.3	6.3	
	3	8.5	18.8	
	4	0	6.3	
	10	0	6.3	
Consumption of folic acid in pregnancy (%)	Yes	11.6	69.2	<0.001
	No	88.4	30.8	
Consumption of dietary supplements	Yes	53.5	15.4	0.015
	No	46.5	84.6	
Use of any water purification system	Water filter	6.8	35.7	<0.001
	Water softener	5.1	21.4	
	No	86.4	42.9	
Consumption of food from PVC containers before sampling	≤24 h	17.5	30.0	0.004
	2 days ago	10.0	50.0	
	>2 days ago	72.5	20	
Consumption of food from PVC bag/film before sampling	≤24 h	20.0	50.0	0.012
	2 days ago	17.5	30.0	
	>2 days ago	62.5	20	
	No	50.0	42.1	
Drinking from plastic sport bottle before sampling	≤24 h	14.0	37.5	0.004
	2 days ago	2.3	37.5	
	>2 days ago	83.8	25.0	
	Multi-apartment home	23.3	25.0	
	Apartment block	30.0	50.0	
	No	96.7	100	

*p*-value—probability value < 0.05 (analysis of variance using one-way ANOVA test for normally distributed data or Kruskal–Wallis test for abnormally distributed data). Legend: EOC—epithelial ovarian cancer; EBOTs—epithelial ovarian borderline tumors.

**Table 4:** Significant correlations between lifestyle habits, determinants of BPA exposure, and urine BPA concentrations.

Total BPA in Urine (µg/L)		Mean/Coeff.	Standard Deviation	*p*-Value
All women (healthy control group + EOC/EBOT group of women)				
Intake of dietary supplements (*n* = 54)	Yes (*n* = 24) No (*n* = 30)	0.21 0.35	0.16 0.28	0.032
Habit of refilling single-use plastic bottle (*n* = 78)	Yes (*n* = 42) No (*n* = 36)	0.32 0.21	0.27 0.14	0.048
Time interval between the last intake of the food from PVC containers to urine sampling (*n* = 48)		−0.327		0.023
Healthy control group				
Habit of refilling single-use plastic bottle (*n* = 78)	Yes (*n* = 31) No (*n* = 28)	0.27 0.17	0.21 0.12	0.051 (*)
Number of amalgam dental fillings (*n* = 28)		0.400		0.035

*p*-value—probability value < 0.05 (analysis of variance using one-way ANOVA for normally distributed data or Kruskal–Wallis test for abnormally distributed data). Legend: BPA—bisphenol A; EOC—epithelial ovarian cancer; EBOT—epithelial ovarian borderline tumors; Coeff.—correlation coefficient; (*)—marginal significance.

## Data Availability

All relevant data are stored by the principal investigator (M.S.) within the University Medical Center Ljubljana, Division of Gynaecology and Obstetrics, and can be shared with other researchers in pseudonymized form if there is an interest.

## Abbreviations

ANOVA	Analysis of variance.
BMI	Body mass index.
BOT	Borderline ovarian tumor.
BPA	Bisphenol A.
BPAF	Bisphenol AF.
BPF	Bisphenol F.
BPZ	Bisphenol Z.
CAT	Catalase
EBOT	Epithelial borderline ovarian tumors.
ECHA	The European Chemical Agency.
EDC	Endocrine-disrupting chemical.
EDTA	Ethylenediaminetetraacetic acid.
EMT	Epithelial–mesenchymal transition.
EOC	Epithelial ovarian cancer.
ER	Estrogen receptor.
FOXO3	Forkhead box O3.
GC-MS/MS	Gas chromatography tandem mass spectrometry.
eGFR	Glomerual filtration rate estimation.
GM	Geometrical median.
GPX1	Gluthatione peroxidase 1.
HBM4EU	European Humam Biomonitoring Initiative.
HBM-GVs	Human biomonitoring guidance values.
IPCS	International Programme on Chemical Safety.
IUPAC	International Union of Pure and Applied Chemistry.
KIKKB	Institute of Clinical Chemistry and Biochemistry.
LOQ	Limit of quantification.
MDRD	Modification of Diet in Renal Disease.
MMPs	Matrix metalloproteinases.
MPV	Mean platelet volume.
MSC	Member State Committee.
NAG	N-acetyl-beta-D-glucosamidase.
NEOC	Non-epithelial ovarian cancer.
OC	Ovarian cancer.
PCOS	Polycystic ovary syndrome.
PVC	Polyvinyl chloride.
RACK1	Receptor for Activated C Kinase 1.
ROS	Reactive oxygen species.
SOD1	Superoxide dismutase 1.
SOD2	Superoxide dismutase 2.
SVHC	Substances of very high concern.
TME	Tumour microenvironment.
USG	Urinary specific gravity.
MDRD	Modification of Diet in Renal Disease.
WHO	World Helath Organization.
